# Process Mechanisms in Behavioral Versus Nondirective Guided Self-help for Parents of Children with Externalizing Behavior

**DOI:** 10.1007/s10578-022-01400-0

**Published:** 2022-09-06

**Authors:** Anne-Katrin Treier, Christopher Hautmann, Christina Dose, Lisa Nordmann, Josepha Katzmann, Julia Pinior, Kristin Katharina Scholz, Manfred Döpfner

**Affiliations:** 1 Department of Child and Adolescent Psychiatry, Psychosomatics and Psychotherapy, Medical Faculty, University of Cologne, Cologne, Germany; 2 School for Child and Adolescent Cognitive Behavior Therapy (AKiP), Medical Faculty, University of Cologne, Cologne, Germany

**Keywords:** Parent training, Self-help, Externalizing behavior, Mediation, Therapist behavior, Adherence

## Abstract

The study examined potential mediating effects of therapist behaviors in the per-protocol sample (n = 108) of a randomized controlled trial comparing a behavioral and a nondirective guided self-help intervention for parents of children with externalizing disorders (4–11 years). Additionally, from an exploratory perspective, we analyzed a sequential model with parental adherence as second mediator following therapist behavior. Outcomes were child symptom severity of attention-deficit/hyperactivity disorder (ADHD) and oppositional defiant disorder rated by blinded clinicians, and parent-rated child functional impairment. We found a significant indirect effect on the reduction of ADHD and functional impairment through emotion- and relationship-focused therapist behavior in the nondirective intervention. Additionally, we found limited support for an extended sequential mediation effect through therapist behavior and parental adherence in the models for these outcomes. The study proposes potential mediating mechanisms unique to the nondirective intervention and complements previous findings on mediator processes in favor of the behavioral group. *Trial registration* ClinicalTrials.gov NCT01350986.

## Introduction

### Efficacy of Parent Training

Parent training has been shown to be effective in the treatment of externalizing behavior disorders in children and adolescents [1, 2]. This pertains to both behavioral and non-behavioral parent training approaches, with neither demonstrating superiority over the other across different outcomes and observers [2, 3]. Parent training is usually implemented face to face, but if there are barriers to face-to-face parent training, e.g., fear of stigma, lack of time, waiting time, or a lack of local treatment options [4], self-help interventions might be a viable treatment alternative [5]. Self-help treatments are psychotherapeutic interventions delivered in written format or via multimedia [6], and range from completely self-administered interventions to guided interventions with additional therapist contact. Especially during the COVID-19 pandemic, self-help interventions were recommended to improve access to psychotherapy [7].

In the treatment of externalizing behavior problems, self-help parenting interventions have demonstrated significant effects on parent-rated parenting behavior, parental wellbeing, child symptoms, and child functional impairment [5, 8, 9]. Notably, the effects on child outcomes emerged despite no direct contact with the child. For blind/observer ratings of child symptoms, the evidence is mixed, with some studies demonstrating effects and others reporting no effects [5, 8]. Additional minimal therapist contact seems to improve the efficacy at least regarding particular outcomes [5, 8]. Guided self-help interventions have demonstrated nearly equivalent effects to face-to-face treatments, but may have the additional advantage of easier accessibility [8].

Regarding different therapeutic approaches, a recent randomized controlled trial compared the efficacy of guided self-help parent training with a behavioral versus a nondirective basis for parents of children with externalizing behavior problems [10]. In both conditions, parents received self-help booklets and telephone consultations. The therapeutic approaches were implemented both through differential contents of the self-help booklets (specific behavior modification strategies in the behavioral group versus focus on parent–child communication in the nondirective group) and through differential instructions for therapeutic behavior in the additional telephone counseling (directive focus on behavior modification in the behavioral group versus reflective focus without specific advice in the nondirective group). Child symptom improvements were found in both groups (e.g., blind-rated ADHD, parent-rated functional impairment), and group differences emerged for specific outcomes in favor of the behavioral group (e.g., blind-rated ODD). However, in line with results on face-to-face parent training, no consistent superiority of either treatment was detected across different outcomes and informants. Moreover, at 12-month follow-up, there were no group differences at all.

### Process Mechanisms of Parent Training

Despite evidence for the efficacy of (self-help) parent training, little is known about the processes responsible for the observed changes. We do not know whether parent training approaches based on different theoretical foundations (e.g., behavioral vs. nondirective approaches) vary in how they induce changes. Understanding these processes is important from a theoretical perspective, and may also help to optimize treatment components [11]. To elucidate mechanisms of change, mediation analyses are typically employed. Mediators are intervening variables that account for the relationship of a dependent variable such as child outcome and an independent variable such as treatment group [11]. In the context of parent training in child and adolescent psychotherapy, the majority of recent mediation studies concentrated on aspects of parent–child interactions, especially facets of parenting behavior, as putative mediators of change [12–14].

### Therapist Behavior as a Mediator of Change

However, little attention has been paid to process-related mediators of change, and knowledge about differential mechanisms of change of behavioral and nondirective parent training is limited. When examining potentially different mechanisms of change between therapeutic approaches, therapist behavior might be of particular interest, as different approaches conceptualize the role and behavior of the therapist in different ways. Several studies highlighted the role of therapist behavior in predicting treatment outcomes in parenting interventions: In a systematic review, Leitao et al. [16] found therapeutic fidelity, structuring of treatment sessions, and positive behavior such as praise to be positively related to parent and child outcomes. In a meta-analysis on behavioral parent management training, Dekkers et al. [17] found that interventions focusing on antecedents of child behavior were positively related to parenting outcomes and psychoeducation was negatively related to parenting outcomes. Barnett et al. [18] even demonstrated a mediation effect of responsive coaching in a behavioral parent training intervention on change in parenting outcomes, while no mediating effect of directive coaching emerged.

While we identified studies showing that therapist behavior in parenting interventions is predictive of treatment outcomes, to our knowledge, there are no mediation studies in the context of parent training based on different therapeutic approaches (e.g., behavioral versus nondirective treatment). Comparing two active intervention groups enables us to investigate differential mechanisms of change. We would expect therapists across all therapeutic approaches to employ basic interpersonal skills such as being empathetic, accepting, and genuine [19]. For behavioral interventions, we would additionally expect a stronger focus on directivity and structures, including contingency management using directive methods such as modeling or homework assignments [2, 20]. For nondirective interventions, we would additionally expect a stronger focus on relationships and emotions, such as guidance on parent-child communication and using interpersonal methods such as facilitating emotional expression [2, 21].

### The Present Study

**Fig. 1:**
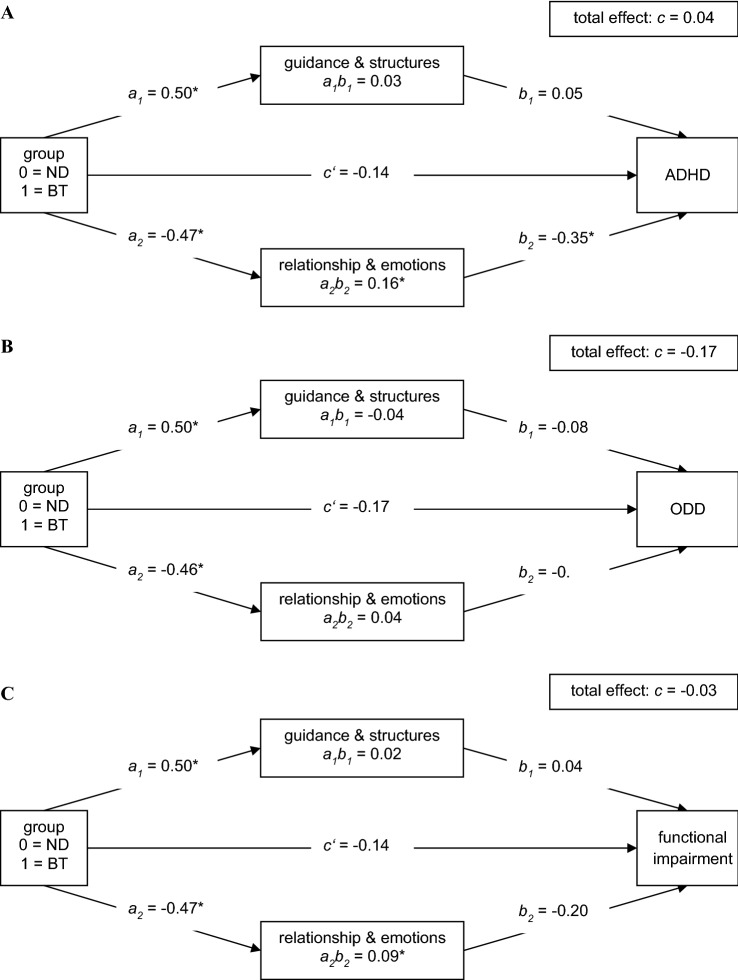
Parallel mediation model for the effects of a nondirective versus a behavioral self-help intervention through therapist behavior (n = 108) on ***a*** ADHD symptom severity ***b*** ODD symptom severity ***c*** functional impairment. Note: Pre-treatment scores of outcomes and single-parent status were included as covariates in the models but are not depicted for the sake of clarity. *BT*  behavioral intervention. *ND* nondirective intervention. *ADHD* attention-deficit/hyperactivity disorder. *ODD* oppositional defiant disorder. *a* unstandardized regression coefficient for the effect of the intervention on a mediator. *b* unstandardized regression coefficient for the effect of a mediator on the outcome. *c*’ unstandardized regression coefficient for the direct effect of treatment on outcome, controlling for putative mediators. *ab* mediation effect. * significant effect

This study aimed to examine therapist behavior as a mediator of the effects of the self-help intervention with a behavioral versus a nondirective basis [see 10] for parents of children with attention-deficit/hyperactivity disorder (ADHD) and oppositional defiant disorder (ODD) on child symptoms of ADHD and ODD, and child functional impairment. We considered these outcome variables since ADHD and ODD are the most important outcome domains for our sample, and functional impairment is a main reason for referral [22]. In particular, we sought to detect differential mediating processes for the two intervention groups. Assuming that parent training exerts its effects on child outcomes indirectly through therapist behavior, we developed and tested the following parallel mediation model (see Fig. 1a–c):

As therapists in the behavioral intervention were instructed to focus directively on teaching the parents specific strategies to deal with the child’s behavior problems, we predicted that they would demonstrate greater guiding and structuring therapist behavior. Even though therapists in both interventions were instructed to counsel in a supportive way, we predicted that the therapists in the nondirective intervention would demonstrate greater emotion- and relationship-focused behavior, as they were specifically instructed to mainly support the parents in reflecting of their feelings and behaviors. Based on previous studies [16–18], we hypothesized that, in turn, high levels of both guiding and structuring therapist behavior and sensitive, emotion- and relationship-focused behavior would lead to a reduction in blind-rated ADHD and ODD symptom severity, and parent-rated functional impairment.

### Parental Adherence as a Potential Sequential Mediator

In recent years, most studies have considered parallel mediators [cf. 12, 13]. However, the complexity of the process may be better reflected by extending the focus to sequential mediation (e.g., study condition affects mediator A, which then influences mediator B, which in turn affects the outcome) [13, 23]. Therefore, we were additionally interested in extending our first model to a sequential mediation model. Although first studies have analyzed sequential mediations in the field of child and adolescent psychotherapy [24, 25], to our knowledge, sequences of mediators have not yet been analyzed in the field of parent training for children with externalizing behavior problems. In parent training, parents’ willingness to actively take part in the intervention and implement the strategies in daily life—parental adherence—might be an essential sequential mediator for the effectiveness of interventions. Common terms for parental adherence are parent involvement or parent (participation) engagement [28]. For our sample, we defined parental adherence as the comprehension of the contents of the self-help booklets and telephone consultations and the implementation of the parenting interventions of the respective treatment group at home. Previous studies found a potential influence of therapist behavior on parental adherence: Leitao et al. [16] reported that empathetic and engaged therapist behavior predicted increased parental adherence [29, 30], while teaching and confronting therapist behavior predicted decreased parental adherence [31]. In a mediation study, Martinez et al. [26] demonstrated that psychoeducation mediated the effect of different treatment approaches on parental adherence. Moreover, there is evidence of a positive relation between parental adherence and treatment outcomes. In their review, Haine-Schlagel and Walsh [28] found consistent evidence of an association of parental adherence with child impairment and inconsistent evidence for child symptoms. Regarding externalizing behavior problems, Kling et al. [27] showed that the effects of behavioral parent training on child symptoms were mediated by homework fidelity.

**Fig. 2:**
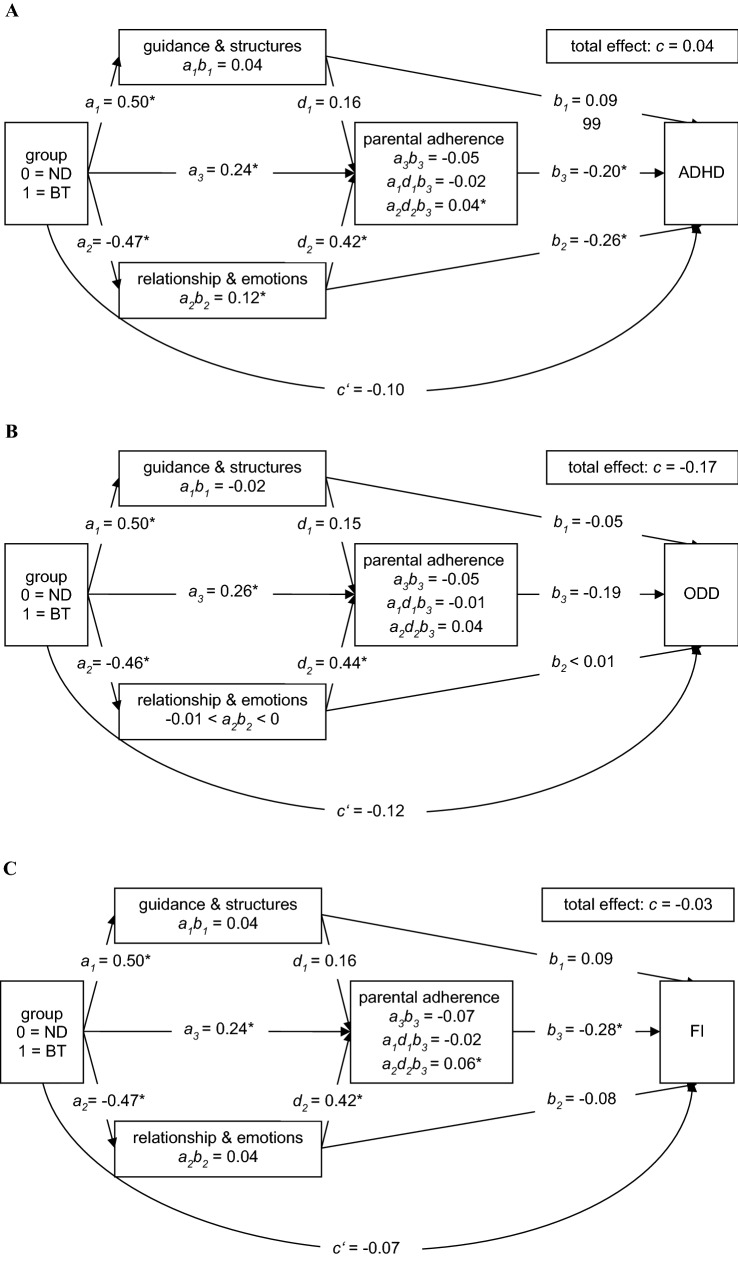
Sequential mediation model for the effects of a nondirective versus a behavioral self-help intervention through therapist behavior (n = 108) on ***a*** ADHD symptom severity ***b*** ODD symptom severity ***c*** functional impairment. Note: Pre-treatment scores of outcomes and single-parent status were included as covariates in the models but are not depicted for the sake of clarity. *BT* behavioral intervention. *ND* nondirective intervention. *ADHD* attention-deficit/hyperactivity disorder. *ODD* oppositional defiant disorder. *FI* functional impairment. *a* unstandardized regression coefficient for the effect of the intervention on a mediator. *b* unstandardized regression coefficient for the effect of a mediator on the outcome. *c*’ unstandardized regression coefficient for the direct effect of treatment on outcome, controlling for putative mediators. *ab* simple mediation effect. abd sequential mediation effect. *Significant effect

Considering these previous findings on parental adherence, we developed and tested the following sequential mediation model from an exploratory perspective (see Fig. 2a–c): As in the previous model, we predicted that therapists in the behavioral intervention would demonstrate a higher level of guiding and structuring therapist behavior, while therapists in the nondirective intervention would demonstrate a higher level of emotion- and relationship-focused behavior. We predicted that, in turn, high levels of both therapist behaviors would lead to increased parental adherence. Finally, we predicted that a higher level of parental adherence would lead to a reduction in blind-rated ADHD symptom severity, blind-rated ODD symptom severity, and parent-rated functional impairment. Due to our limited sample size, this extension of the model was considered as exploratory.

## Method

### Study Design

Data were taken from the randomized controlled trial by Hautmann et al. [10]. Families were allocated to a guided self-help intervention with either a behavioral or a nondirective basis using block randomization. Parents in both treatment groups received eight self-help booklets fortnightly by mail, and ten fortnightly telephone consultations with a therapist, each scheduled to last about 20–30 min. The final two consultations were booster sessions. The telephone consultations were audiotaped if permitted by the parents (permission granted by 108 of 110 families). Data were collected before the beginning of the intervention (pre-treatment), during treatment (mediators), and after the 5-month intervention period (post-treatment). For details of the full procedure, see Hautmann et al. [10].

### Participants

#### Families

Families were recruited through institutions of the local health care systems (e.g. pediatricians, counseling services) throughout Germany. The institutions registered the families, who were then contacted by the researchers. Inclusion criteria were child age of 4–11 years and a child diagnosis of ADHD and/or ODD according to the DSM-IV [32] based on clinical interview [Disorder-specific diagnostic checklists of the Diagnostic system for psychiatric disorders in children and adolescents according to ICD-10 and DSM-IV [DISYPS-II]; [33] with the parent(s) conducted by telephone. Exclusion criteria encompassed an indication of an intellectual disability (clinical evaluation by local health provider) or a diagnosis of an autism spectrum disorder (telephone screening interview), indication of the need for more intensive treatment, an existing psychotherapy with a focus on parent training, a planned change in medical treatment, insufficient motivation to participate in the study, and insufficient German language skills. The study was approved by the Medical Ethics Committee of the University Hospital of Cologne. Informed consent was obtained from all parents prior to inclusion in the study.

While 149 parents were randomized (intention-to-treat sample), 51 parents in the behavioral and 59 parents in the nondirective intervention completed the treatment (i.e., received eight booklets and participated in ten telephone consultations; per-protocol sample). For parents who dropped out prior to completion, no or only partial mediator and outcome data were available. Therefore, the present study focused on the per-protocol sample. Two families did not provide permission to audiotape the telephone consultations, resulting in a final sample of 108 parents.

#### Therapists

The telephone consultations were performed by five therapists (degree in psychology or pedagogy) who were in training to become child and adolescent behavioral psychotherapists. All therapists treated parents in both treatment arms. To promote treatment integrity, several measures were implemented: Therapists received intensive training in both interventions and supervision was conducted regularly by experienced psychotherapists with specific training in the behavioral or nondirective treatment approach, integrating samples of audiotaped sessions.

### Self-help Booklets

The self-help booklets for the behavioral intervention were developed based on a behavioral self-help book [34] addressed at parents of children with externalizing behavior problems [35]. The booklets contained psychoeducational information about externalizing behavior problems, guidance on problem definition, the analysis of specific behavior problems and guidance on behavioral interventions such as promoting of positive parent–child interactions, reviewing and implementing family rules, effective commands, positive and negative consequences, and promoting the child’s strengths. For the implementation of the interventions in daily life, the behavioral booklets contained worksheets, “memo cards” with the most important take-home messages, and homework assignments.

The self-help booklets for the nondirective intervention were based on a nondirective self-help book [36] targeting challenging situations for parents in general [37]. The booklets contained psychoeducational information about parent–child interactions, guidance on demonstrating acceptance towards the child, and information on nondirective interventions such as active listening, I-messages and joint conflict resolution.

### Measures

#### Therapist Behavior

The extent to which therapists demonstrated (1) guiding and structuring behavior and (2) relationship- and emotion-focused behavior in the audiotaped counseling sessions was rated by blinded clinicians using the Therapist Intervention Scale—Therapist Behavior [TIS-Therapist, 38]. The rating scale comprises the subscales *Guidance & Structures* (10 items) and *Relationship & Emotions* (7 items). The first subscale contains items on guiding and structuring therapist behavior, including guidance for the management of specific problem situations (e.g. “The therapist defines or analyzes the problem based on a specific situation together with the parent”, “The therapist guides the parent to use positive reinforcement (verbally and nonverbally) in regard to the child’s positive behavior or characteristics” or “The therapist assigns homework”). The second subscale contains items on therapeutic interventions with a focus on exploring and expressing feelings, and building relationships (e.g. “The therapist encourages the parent to recognize and express positive and negative feelings towards the child”, “The therapist encourages the parent to perceive and understand the feelings of the child”, “The therapist expresses unconditional positive regard and acceptance for the parent”). All items were rated on a 3-point Likert-type scale ranging from 0 (*not at all*) to 2 (*extensively*).

For each family, one audiotaped telephone counseling session was randomly selected, stratified by treatment phases in the ratio of 2:6:2 to ensure that all intervention periods were represented according to the number of sessions [39]: psychoeducation (sessions 1–2), intervention (sessions 3–8), booster (sessions 9–10). For the analyses, the mean item score per subscale was calculated. In the present sample, the TIS-Therapist demonstrated good to excellent interrater reliability based on 20 randomly selected double-rated audiotaped sessions, with values of 0.91 and 0.71 for the subscales *Guidance & Structures* and *Relationship & Emotions*, respectively [ICC[1,2]; 38]. Furthermore, based on McDonald’s Omega [40], both subscales demonstrated an acceptable to good internal consistency, with values of 0.85 and 0.71 for the subscales *Guidance & Structures* and *Relationship & Emotions*, respectively [38].

#### Parental Adherence

The therapist-rated parental adherence was measured with the self-developed 2-item clinical rating scale *Parental Adherence (P-ADH)* after each telephone consultation. One item assessed the comprehension of the information given in the booklets and by the therapists (“On an overall basis, to what extent did the parent comprehend the content?”) and one item assessed the implementation of the treatment components in the parents’ daily practices (“To what extent did the parent implement the interventions?”). Both items were rated on a 3-point Likert-type scale ranging from 0 (*poor*) to 2 (*good*). The rating was based on the clinical impression during the counseling sessions and the information given by the parent: Therapists were instructed to ask parents during each session how well they comprehended the instructions, how helpful the strategies were, and how often they implemented the strategies. Therapists completed the scale after each of the first eight telephone sessions, in which parents had received new input through the booklets.

As we aimed to include ratings of therapist behavior from all treatment phases (i.e. sessions one–ten), for the exploratory sequential mediation model, we were therefore unable to assess the mediators chronologically for all families. However, to incorporate the idea of the hypothesized sequential model, we chose to use only the last four rated sessions of parental adherence. Thus, we calculated the mean item score of sessions five to eight. In the present study, the P-ADH demonstrated good internal consistency, with a value of 0.87 based on McDonald’s Omega [40].

#### Externalizing Behavior

ADHD and ODD symptom severity were rated by blinded clinicians using the *Diagnostic Checklist for Attention Deficit/Hyperactivity Disorder* (DCL-ADHD) and, due to the age range of the sample, the oppositional-aggressive subscale of the *Diagnostic Checklist for Disruptive Behavior Disorders* (DCL-DBD ODD), which are part of the German diagnostic system *DISYPS-II* [33]. Ratings were based on audiotaped semi-structured clinical interviews by the respective therapists. To ensure blinding, any information referring to intervention group or time of assessment was erased prior to the blinded rating. The DCL-ADHD comprises 18 items while the DCL-DBD ODD comprises eight items. All items were rated on a 4-point Likert-type scale ranging from 0 (*age-appropriate*) to 3 (*extensively*). Blinded clinicians completed the checklists at pre-treatment and post-treatment. For the analyses, the mean item score per scale was calculated. The DCL-ADHD and DCL–DBD ODD have demonstrated excellent interrater reliabilities of 0.94 and 0.98, respectively [ICC[2,2]; see 10]. In the present study, both scales demonstrated good internal consistencies, with values of 0.85 and 0.80 for the DCL-ADHD and values of 0.78 and 0.79 for the DCL-DBD ODD at pre- and post-treatment, respectively, based on McDonald’s Omega [40].

#### Functional Impairment

Children’s functional impairment was rated by parents using a German adaptation of the parent form of the *Weiss Functional Impairment Rating Scale* [WFIRS-P; 41, 42]. The total scale comprises functional impairment in the domains of family, learning and school, life skills, child’s self-concept, and social activities. The 40 items were rated on a 4-point Likert-type scale ranging from 0 (*not at all*) to 3 (*extensively*). Parents completed the scale at pre- and post-treatment. For the analyses, the mean item score was calculated. In the present study, the WFIRS-P demonstrated excellent internal consistency, with values of 0.92 and 0.95 at pre- and post-treatment, respectively, based on McDonald’s Omega [40].

### Statistical Analyses

We applied a Bayesian stochastic regression imputation approach for single missing data, as this approach is able to account for uncertainty of the predicted values by considering error variance [43]. For the imputation, we considered sociodemographic variables, baseline data, process variables, and data at post-treatment as predictors. Missing data was 13% maximum for all variables except for adherence, with a higher percentage of 33%. Missing data analyses with Little’s MCAR test [44], including all variables relevant for the mediation models, indicated that data were missing completely at random: χ²(61) = 64.54; *p* = .354.

To test for baseline group differences, demographic and baseline data were compared between the treatment groups using Chi-square tests for categorical variables and independent samples *t*-tests for continuous variables. To examine whether the effects of the behavioral versus the nondirective self-help intervention on ADHD symptom severity, ODD symptom severity, or functional impairment, respectively, were mediated by therapist behavior and parental adherence, we performed mediation analyses. For this purpose, we employed the PROCESS macro for SPSS [45] developed by Hayes [46], which uses ordinary least squares regression to estimate the model parameters.

In a simple mediation model, an independent variable *X* exerts its effects on a dependent variable *Y* indirectly through a mediating variable *M*. The total effect (*c*) of *X* on *Y* comprises a direct effect (*c*’) and an indirect effect through *M* [ab; 47]. The direct effect is the effect of *X* on *Y* when controlling for *M* [46]. The indirect effect is the product of the effect of *X* on *M* – *a* – and of the effect of *M* on *Y* – *b* – [47].

When considering several possible mediators, it is recommended to examine them together in a multiple mediation model [46]. If mediators are assumed to not causally influence each other, they can be modeled as parallel mediators. In a sequential multiple mediator model, on the other hand, one mediator is assumed to cause changes in another mediator [46]. Thus, in a sequential mediation model with two mediating variables *M*_*1*_ and *M*_*2*_, there is an indirect effect through *M*_*1*_ and *M*_*2*_ (*adb*) in addition to the direct effect (*c*’) and the specific indirect effects of *M*_*1*_ and *M*_*2*_ [ab; 47].

In the present study, we examined the hypothetical model that the treatment group would lead to different levels of guiding and structuring as well as emotion- and relationship-focused therapist behavior, which would then cause changes in treatment outcomes (i.e., ADHD symptom severity, ODD symptom severity, or functional impairment). Thus, we considered treatment condition as independent variable and treatment outcome as dependent variable and modeled the aforementioned aspects of therapist behavior (as captured by the two TIS-Therapist scales) as parallel mediators (model number 4 in PROCESS, see Fig. 1a–c). For the additional analysis of the sequential models, we examined, from an exploratory perspective, whether therapist behavior would cause changes in parental adherence, which would in turn lead to changes in the treatment outcomes (model number 80 in PROCESS, see Fig. 2a–c).

We tested the model separately for each outcome measure (post-treatment ADHD symptom severity, post-treatment ODD symptom severity, post-treatment functional impairment). It should be noted that contrary to earlier recommendations [48], the current literature does not consider a non-significant main effect to be an obstacle to mediation analyses [46]. Following Hayes [46], we included the pre-treatment score of the respective outcome variable as covariate. As there was a higher percentage of single parents in the nondirective treatment arm than in the behavioral treatment arm (see Table 1), we included this variable as an additional covariate in the model. The inclusion of this variable might be of special importance in the sequential mediation model, as the adherence to a self-help program might me more challenging for single-parent families.

For the interpretation of indirect effects, we focused on the product of the effects constituting these indirect effects instead of considering the significance of the single paths defining them [46]. That is, we considered the products *a*_1_*b*_1_ and *a*_2_*b*_2_ to evaluate the presence of mediation effects in the parallel mediator model and the products *a*_1_*d*_1_*b*_3_ and *a*_2_*d*_2_*b*_3_ to evaluate the presence of sequential mediation effects. As recommended, we report unstandardized regression coefficients and used bias-corrected bootstrapping with 5000 resamples to estimate confidence intervals [46]. Effects were classified as significant if the 95% confidence intervals did not include zero. Moreover, we considered partially standardized coefficients for the mediation effects (effects relative to the standard deviation of the dependent variable) to gain an impression of the effect size, and considered the proportion of outcome variance explained by each model (*R*²) to examine its particular goodness of fit.

## Results

### Sample Characteristics

Sample characteristics are displayed in Table 1. The children’s mean age was 7.19 years (*SD* = 1.98) and 80% were male. 75% met the diagnostic criteria for ADHD and 79% met the diagnostic criteria for ODD. Thus, 54% met the criteria for both diagnoses. The mean age of the participating parents was 38.44 years (*SD* = 6.91) and 97% were female. There were no significant differences between the groups regarding age and gender of the child or the participating parent, the problem behavior of child, the functional impairment of the child, or the number of years of education of the participating parent. However, significantly more single parents participated in the nondirective self-help intervention (see Table 1).

**Table 1:** Demographic and clinical characteristics at pre-treatment and tests for between-group differences

Variable	Behavioral self-help intervention (*n* = 51)			Nondirective self-help intervention (*n* = 57)			Test statistic
	*M*	SD	%	*M*	SD	%	
Child variables							
Age (years)	7.06	1.89		7.32	2.07		*t*(106) = 0.67
Gender (male)			82.4			77.2	χ²(1) = 0.44
ADHD	1.47	0.48		1.42	0.58		*t*(106) = 0.49
ODD	1.40	0.61		1.42	0.50		*t*(106) = 0.17
FI	0.89	0.40		0.95	0.44		*t*(106) = 0.79
Parent variables							
Age (years)	38.14	7.40		38.72	6.49		*t*(106) = 0.44
Gender (female)			96.1			98.2	χ²(1) = 0.47
Education (years)	12.75	2.61		12.91	2.89		*t*(106) = 0.31
Single-parent status			9.8			24.6	χ²(1) = 4.04*

Years of education were calculated based on the ISCED-97 classification

*ADHD* symptoms of attention-deficit/hyperactivity disorder (rated by blinded clinician), *ODD*  symptoms of oppositional defiant disorder (rated by blinded clinician). *FI* functional impairment (parent-rated)

*p < .0.05 (not adjusted)

### Parallel Mediation Models

The results for the parallel mediation models (including ADHD symptom severity, ODD symptom severity, or functional impairment as dependent variable) are presented in Fig. 1a–c. We identified a significant indirect effect of group on post-treatment ADHD symptom severity and functional impairment through emotion- and relationship-focused therapist behavior in favor of the nondirective group (*a*_2_*b*_2_). That is, the nondirective treatment was associated with a higher level of emotion- and relationship-focused therapist behavior, which was in turn associated with lower levels of post-treatment ADHD symptom severity and post-treatment functional impairment. The respective significant partially standardized indirect effects through emotion- and relationship-focused therapist behavior were 0.41 in the model considering ADHD symptom severity as outcome and 0.19 in the model considering functional impairment as outcome. The non-significant partially standardized indirect effect in the model using ODD symptom severity as outcome was 0.07. In other words, on average, two patients from different treatment groups differ by about 41%, 19%, and 7% of a standard deviation in their ADHD, functional impairment, and ODD scores, respectively, because of the indirect effects through relationship- and emotion-focused behavior.

Moreover, guiding and structuring therapist behavior could not be established as a mediator. The non-significant partially standardized effects were 0.07 for ADHD symptom severity, − 0.08 for ODD symptom severity, and 0.04 for functional impairment.

The parallel mediation models comprising the treatment group, the mediators, and the covariates explained 29% of the variance in ADHD symptom severity, 25% of the variance in ODD symptom severity, and 42% of the variance in functional impairment.

### Exploratory Analysis of Sequential Mediation models

The results for the sequential mediation models (including ADHD symptom severity, ODD symptom severity, or functional impairment as dependent variable) are presented in Fig. 2a–c. We identified a significant sequential indirect effect of group on post-treatment ADHD symptom severity and functional impairment through emotion- and relationship-focused therapist behavior and parental adherence (*a*_2_*d*_2_*b*_3_). That is, the nondirective treatment was associated with a higher level of emotion- and relationship-focused therapist behavior, which was in turn associated with increased parental adherence, and finally led to reduced post-treatment ADHD symptom severity and functional impairment. The respective significant partially standardized indirect effects lay at 0.10 for the model including ADHD symptom severity as outcome and 0.11 for the model considering functional impairment as outcome. The non-significant partially standardized indirect effect in the model using ODD symptom severity as outcome was 0.08.

Guiding and structuring therapist behavior could not be established as part of a sequential mediating process. The partially standardized effects were − 0.04 for ADHD symptom severity, − 0.03 for ODD symptom severity, and − 0.05 for functional impairment.

The models including the sequential mediation comprising the treatment group, the mediators, and the covariates explained 33% of the variance in ADHD symptom severity, 27% of the variance in ODD symptom severity, and 48% of the variance in functional impairment.

## Discussion

The present study extends the research on process mechanisms by analyzing differential mediating mechanisms in a guided self-help intervention for parents of children with externalizing behavior disorders with a behavioral versus a nondirective basis. When controlling for baseline levels, we found a significant indirect effect on both child ADHD symptoms and functional impairment through emotion- and relationship-focused therapist behavior in favor of the nondirective intervention. Additionally, we found a sequential mediation effect through emotion- and relationship-focused therapist behavior and parental adherence in the models for these outcomes in our exploratory analyses.

Previous literature reported a link between positive or responsive therapeutic behavior and improved treatment outcomes [16, 18]. In accordance with these findings, our results revealed a significant mediation effect through emotion- and relationship-focused behavior. However, this effect only emerged for the nondirective intervention. As mentioned above, we expect therapists across different therapeutic approaches to employ basic interpersonal skills such as being empathetic, accepting, and genuine [19]. Nevertheless, therapists in nondirective interventions tend to address these interventions in a more intensive and sustained manner, both in their behavior and in the therapeutic content, e.g., by giving guidance on supportive parent-child communication [2, 21]. Therefore, in line with our hypotheses, therapists in the nondirective group demonstrated more emotion- and relationship-focused behavior than therapists in the behavioral group. Consistent with the theory underlying the nondirective approach, emotion- and relationship-focused behavior was associated with improved symptoms and impairment. To induce change, therapists might therefore have to focus more intensively and more explicitly on emotion- and relationship-focused behavior.

Contrary to our expectations, we found no evidence for the role of guiding and structuring behavior as a mechanism of change in favor of the behavioral intervention group as compared to the nondirective group. Our expectations were based on previous findings that structuring behavior and a focus on antecedents were related to improved treatment outcomes [16, 17]. However, in line with the current results, Barnett et al. [18] only demonstrated a mediation effect through responsive behavioral coaching but not through directive behavioral coaching. Interestingly, the authors also provided an explanation for this pattern of parents’ skills demonstrated within the session, reporting that parents with fewer skills were coached in a more directive manner. Thus, directive therapist behavior might be confounded with parental skills. Future research might therefore assess and analyze parental skills as a covariate of the proposed mediation model.

Our additional exploratory analyses suggest that there might even be a sequential mediation process in the models for ADHD and functional impairment. In particular, a more emotion- and relationship-focused behavior of the nondirective therapist might have improved parents’ ability and willingness to engage in therapy, which might then have led to a symptom reduction in the child. This finding is in line with previous research demonstrating that empathetic and engaged therapist behavior predicted parental adherence [29, 30] and that parental adherence predicted at least some treatment outcomes [27, 28]. As this is the first study to suggest a sequential mediation model for the mediation of the effects of parent training on externalizing behavior, future research should further analyze and potentially replicate the effect. If the proposed sequential mediation effect can be replicated, this may imply that emotion- and relationship-focused therapist behavior in nondirective interventions is particularly helpful for parents at risk of low parental adherence, such as those with lower socioeconomic status or parental mental health problems [28], to improve both adherence and treatment outcomes. Furthermore, it would be interesting to extend the definition of parental adherence to attendance of sessions. As we focused on parents who fully completed the intervention, we were unable to include this factor in our analyses.

Interestingly, the specific mediation effect through emotion- and relationship-focused behavior was stronger in the parallel mediation model considering ADHD symptoms as outcome than in the parallel mediation model considering functional impairment as outcome. This was surprising given previous suggestions that environmental factors might play a more pronounced role in the development of functional impairment and ODD symptoms than in the development of ADHD symptoms [49, 50]. Therapists’ empathetic and accepting behavior, combined with the encouragement to express feelings, might have led to relief and an acceptance of negative feelings and behaviors both in the parents and in their child. Additionally, parents in the nondirective intervention might have communicated with their child more empathetically and supportively. ADHD core symptoms potentially result from a motivational dysfunction, and children with ADHD respond particularly strongly to social rewards [51]. Thus, parents’ more supportive communication with their children following therapists’ emotion- and relationship-focused behavior in the nondirective intervention might have contributed to the stronger mediation effect in the ADHD model.

To gain an impression of the model fit of our models, we analyzed the proportion of variance in the outcome variables explained by treatment group, the covariates, and the mediators together. We were able to explain a substantial proportion of the variance at post-assessment with our parallel mediation models that is a quarter to a third of the variance in ADHD and ODD symptom severity and between 42% and 48% of the variance in functional impairment. Thus, although our models explained a considerable amount of variance, there is still scope to examine further process mechanisms. In our models, pre-treatment scores of functional impairment seemed to be particularly important for post-treatment scores as compared to ADHD and ODD symptom severity, indicating that functional impairment might have been more stable than child symptoms. This finding is in line with previous research demonstrating that the assessment of improvements in child externalizing symptoms during treatment might fail to consider continued problems in functioning [52]. The higher stability of functional impairment might therefore indicate that some contributing factors were not targeted within our interventions. Since both interventions focused on parent–child interactions, impairment such as in school or with peers, or impairment due to comorbidities, might consequently not have improved as much.

Analyzing the same data as in the present study, Katzmann et al. [15] found a mechanism of change specific to the behavioral program, and showed that the behavioral program exerted its positive effects on child behavior problems through an improvement in parental attributions. The present findings and those from Katzmann et al. [15] can be seen in a complementary fashion, with one analysis showing a specific mediating mechanism in favor of the behavioral program and the other in favor of the non-behavioral program. This corresponds to the idea of different or even opposing mediation effects leading to similar outcomes in both treatment approaches. To interpret the present findings, it is important to emphasize that our study design does not allow us to identify shared processes, as we did not include an untreated control group. These shared processes might have played a role, as there were several similarities across the two interventions, such as the focus on improving parent–child interactions or the instruction for therapists to counsel in a supportive way.

Some limitations to the present findings should be mentioned. First, all therapists were in training to become behavioral therapists, but counseled families in both interventions. Accordingly, therapists might have shown greater expertise in the behavioral treatment and, additionally, might have identified themselves more with the behavioral program [allegiance effect; 53]. To promote a comparable treatment integrity in the two groups, we took numerous actions, such as intensive training, regular supervision with experts in their field, or sample audiotapes to monitor therapist behavior. Through therapists performing therapies in both intervention groups, we intended to minimize the influence of unique therapist characteristics, thereby making the interventions more comparable [54].

The second limitation lies in our implementation of the blinded rating. As mentioned above, blinded ratings were based on structured interviews with the participating parent(s), and no direct exploration or observation of the child was conducted. Instead of depicting actual changes in child behavior, the ratings of post-treatment ADHD symptoms may rather reflect a change in the parents’ evaluation of their child’s behavior. Direct observation of child behavior should be used in future studies to test the validity of our findings.

Third, the parents in our sample had a rather high level of education (almost 13 years). The ability to structure the learning process and the implementation of changes at home might be especially crucial for self-help interventions. Thus, parents with higher levels of education might be more willing to participate in and complete self-help interventions. This notion is in line with studies indicating a higher likelihood of early treatment termination for parents with lower education in face-to-face training [55]. Our results might therefore not be generalizable to parents with lower educational levels.

Fourth, there are some limitations specific to the sequential mediation model. As mentioned above, due to our limited sample size, the analysis of the sequential mediation model was considered exploratory in nature. For the parallel mediation model, the required sample size to detect moderate or small to moderate mediation effects is between 77 and 115 [56]. However, for a more complex model such as a sequential mediation model, a larger sample size is needed to be able to detect the same effects.

Furthermore, in order to draw causal inferences, it is important to determine a timeline for the components of the mediation process [11]. As stated above, in some families, a chronological assessment of the mediators in correspondence with their chronological appearance in the sequential models could not be established. To examine the possibility of a reverse order of the mediator sequence, we calculated a mediator model with parental adherence as the first mediator and therapist behavior as a subsequent mediator, and the results indicated no sequential mediation. This finding, in combination with our theoretical model and previous studies demonstrating that therapist behavior predicted parent engagement [16, 26], increases the likelihood of the assumed causal order.

Additionally, there was a high percentage of missing data for the adherence ratings. However, when analyzing only cases without any missing data, the effect sizes of the detected sequential mediation effects were at least comparable. Even though the sample size was smaller, the effect was still significant in the model including functional impairment as outcome, while this was not the case in the model including ADHD as dependent variable. Taken together, these limitations of the sequential models indicate that the associated findings should be interpreted with caution.

Our findings contribute to the understanding of which mechanisms of change are unique and effective to a particular treatment approach. To our knowledge, this is the first study to analyze differential aspects of therapist behavior as well as sequential mediation in the context of parent training for externalizing problem behavior. Our results indicate that the stronger focus on an emotion- and relationship-focused therapist style in the nondirective intervention might have led to a reduction in ADHD symptom severity and functional impairment in the child, potentially by encouraging parents to adhere to the treatment. This highlights the role of emotion- and relationship-focused behavior for the induction of changes. No specific mechanism of change was revealed for the intervention with a behavioral basis. However, this might be due to the sample size and the limited scope of the mediators under investigation. Previous findings have demonstrated mechanisms of change unique to the behavioral program as compared to the nondirective intervention [15]. Further research could integrate the different results into a more general model with a larger sample size. We consider these findings particularly important and potentially generalizable given that the interrelations were established across domains rated by different informants. In sum, the study proposes potential mediating mechanisms unique to the nondirective intervention. To gain a deeper understanding of how interventions with different theoretical foundations vary in how they induce change, further research is needed. Only if we understand the processes responsible for change can we optimize treatment components adequately.

## Summary

The present study examined differential process mechanisms of a guided self-help parent training targeting externalizing behavior with a behavioral versus a nondirective basis. In a randomized controlled trial, a behavioral and a nondirective guided self-help parent training intervention were compared in a sample of 4–11-year-old children with externalizing disorders. Possible mediating effects of therapist behaviors (guiding and structuring behavior, emotion- and relationship-focused behavior) were examined in the per-protocol sample (n = 108) using regression analyses. Additionally, the model was extended, in an exploratory manner, to a sequential mediation model with parental adherence as second mediator following therapist behavior. Outcomes were child symptom severity of attention-deficit/hyperactivity disorder and oppositional defiant disorder rated by blinded clinicians, and parent-rated child functional impairment. We did not identify process mechanisms for the intervention effects on oppositional defiant disorder or in favor of the behavioral intervention, potentially due to the sample size and the limited scope of the mediators under investigation. We detected a significant indirect effect through emotion- and relationship-focused therapist behavior on the reduction of ADHD and functional impairment in favor of the nondirective intervention. This highlights the role of emotion- and relationship-focused behavior for the induction of changes. Additionally, exploratory analyses yielded some support for a potential sequential mediation effect through emotion- and relationship-focused therapist behavior and parental adherence in the models considering ADHD symptoms and functional impairment as outcomes. The findings suggest potential mediating mechanisms unique to a nondirective self-help parent training intervention and complement previous findings on mediator processes in favor of the behavioral group. Future studies should replicate the proposed differential mechanisms of change in a comprehensive and integrative model with a larger sample. To gain a deeper understanding of how interventions with different theoretical foundations vary in the way they induce change, further research is needed. Only if we understand the processes responsible for change, we can optimize treatment components adequately.
